# Endocytic Trafficking at the Mature Podocyte Slit Diaphragm

**DOI:** 10.3389/fped.2017.00032

**Published:** 2017-02-24

**Authors:** Agnieszka Swiatecka-Urban

**Affiliations:** ^1^Department of Nephrology, Children’s Hospital of Pittsburgh, Pittsburgh, PA, USA; ^2^Department of Cell Biology, University of Pittsburgh School of Medicine, Pittsburgh, PA, USA

**Keywords:** nephrotic syndrome, proteinuria, endocytic trafficking, endocytosis, podocytes, nephrin, podocin, glomerular slit diaphragm

## Abstract

Endocytic trafficking couples cell signaling with the cytoskeletal dynamics by organizing a crosstalk between protein networks in different subcellular compartments. Proteins residing in the plasma membrane are internalized and transported as cargo in endocytic vesicles (i.e., endocytosis). Subsequently, cargo proteins can be delivered to lysosomes for degradation or recycled back to the plasma membrane. The slit diaphragm is a modified tight junction connecting foot processes of the glomerular epithelial cells, podocytes. Signaling at the slit diaphragm plays a critical role in the kidney while its dysfunction leads to glomerular protein loss (proteinuria), manifesting as nephrotic syndrome, a rare condition with an estimated incidence of 2–4 new cases per 100,000 each year. Relatively little is known about the role of endocytic trafficking in podocyte signaling and maintenance of the slit diaphragm integrity. This review will focus on the role of endocytic trafficking at the mature podocyte slit diaphragm.

## Mechanisms of Endocytic Trafficking

All living cells process information by trafficking cargo from the plasma membrane in endocytic vesicles (i.e., endocytosis) and by returning much of the internalized membrane to the cell surface by a reciprocal process called recycling. The balance between endocytosis and recycling controls the plasma membrane composition and provides cells with information that has been resolved in time and space. Endocytic trafficking controls the supply of adaptor proteins and cargo, sorts the internalized cargo to specific intracellular compartments, and orchestrates the crosstalk in intracellular vesicles (1–3). As a result, cells turn over the equivalent of the entire plasma membrane one to five times per hour. Although endocytosis and recycling are ubiquitous, specific endocytic motifs and an assortment of protein adaptors guide cargo to diverse trafficking pathways. Defective endocytic trafficking has been associated with human disease, including congenital malformations, cancer, inflammation, and immunodeficiency (1). The mechanisms of endocytic entry of protein cargo, endocytic compartments, and mechanisms of cargo recycling were recently reviewed and will not be discussed here (4).

## Endocytic Trafficking at the Mature Podocyte Slit Diaphragm

**Figure 1:**
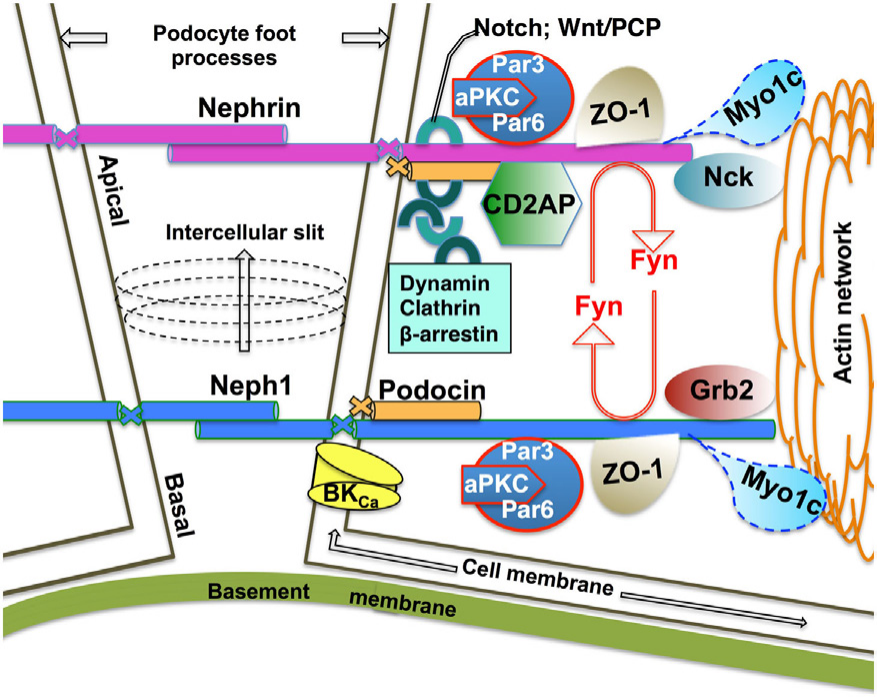
**Schematic of the functional Neph1–nephrin protein complex at the mature mammalian slit diaphragm**. Homophilic interactions of the integral membrane proteins nephrin and Neph1 form the mature mammalian slit diaphragm with a ratio of 1:2.5, respectively (10). Nephrin homophilic interactions are located more apically while the Neph1 strands localize more basally at the intercellular slit (10). The number of Ig repeats in the extracellular domain (9 and 5 for nephrin and Neph1, respectively) determines, at least in part, the width of the intercellular slit (10). The PHB domain-containing protein podocin anchors nephrin and Neph1 at the cell membrane. The intracellular domain of nephrin and Neph1 interacts with the cortical actin cytoskeleton *via* the juxtaposed cytoplasmic protein networks of junctional scaffolding proteins, channels, adaptor proteins, including endocytic adaptors, protein kinases, and motor proteins. Fyn-mediated phosphorylation of nephrin and Neph1 is essential for the podocyte architecture and signaling. Growing evidence supports a model where endocytic trafficking of the integral membrane proteins dynamically controls podocyte architecture and signaling. While the endocytic itineraries of nephrin are better understood, very little is known about the Neph1 endocytic mechanisms. Published data demonstrate that the motor protein Myo1c recruits Neph1 to the podocyte membrane and controls the cell membrane turnover of Neph1 in an actin-dependent manner (20, 21).

The kidney glomerulus is a filtering apparatus that allows passage of water and solute into the urinary space while retaining the vast majority of plasma proteins within the circulation. The functional unit of glomerular filtration is formed by the epithelial cells podocytes, glomerular basement membrane (GBM), and fenestrated capillary endothelial cells (5). The mature podocyte consists of the cell body, primary and secondary processes with microtubule and intermediate filament-based cytoskeleton, and the actin-based foot processes. Resting on the GBM, foot processes form interdigitating extensions linked by the slit diaphragm, which provides the only cell–cell contact between mature podocytes (6). Slit diaphragm is critical for (i) providing selective permeability between the blood and urinary space, (ii) separating the apical and basolateral plasma membrane, and (iii) serving as a signaling platform (4). Structurally, a network of proteins residing at the slit diaphragm membranes connects with the actin cytoskeleton through a juxtaposed cytoplasmic protein scaffold (7). Strong evidence demonstrates that the slit diaphragm is a dynamic unit (8, 9), suggesting that endocytic trafficking of membrane proteins plays an integral role in regulating the dynamics (Figure 1). Recent data provided genetic, functional, and high-resolution ultra-structural evidence highlighting a model of dynamic and multilayered architecture of the slit diaphragm (10). According to the model, the mammalian intercellular slit has a specific arrangement of the integral membrane proteins nephrin and Neph1 at a ratio of 1:2.5. The homophilic interactions between extracellular domains of nephrin are formed more apically, while the homophilic interactions of Neph1 localize closer to the basement membrane at the intercellular slit (10). Difference in the length of extracellular domain of nephrin and Neph1 results in a different length of the intercellular strands formed by the homophilic interactions that subsequently determine the width of the slit (10). Nephrin is an immunoglobulin-type cell adhesion molecule critical to the slit diaphragm function (11, 12). Mutations in the *Nphs1* gene encoding nephrin protein lead to congenital nephrotic syndrome characterized by absence or profound impairment of the slit diaphragm and manifesting as severe proteinuria or nephrotic syndrome (13, 14). Although nephrin was shown to undergo endocytosis (15–19), relatively little is known how this process dynamically controls the slit diaphragm integrity. Data reviewed below demonstrate current understanding of the protein–protein interactions that regulate nephrin endocytic trafficking. The role of Neph1 is discussed in a separate section.

Nephrin localization at the slit diaphragm is determined by podocin, a lipid raft-resident protein encoded by the *Nphs2* gene (22, 23). The prohibitin homology (PHB) domain—a lipid recognition motif present in podocin may target the podocin–nephrin complex to the lipid raft domains of slit diaphragm membranes (24, 25). Certainly, the *Nphs2* gene mutations that mislocalize podocin also mislocalize nephrin and lead to steroid resistant, hereditary, and sporadic nephrotic syndrome (22, 26). The podocin C-terminal T^339^VV motif regulates the cell surface localization as well as the lipid raft-independent podocin stability (27). While tagged podocin resides specifically in the late endosomal/lysosomal compartment (27), mislocalized endogenous podocin co-localizes with an early endosomal marker Rab5 in the puromycin nephrosis rat model (28). Despite the above data, a large gap in understanding of the podocin endocytic itineraries still exists. Flotillins are members of the PHB domain-containing protein family. PHB domains contain hairpin-forming, hydrophobic regions that help to insert a protein into the inner leaflet of plasma membrane. The PHB domain and a region upstream of the N-terminus, modified posttranslationally by palmitoylation and myristoylation, mediates binding of flotillins to the inner leaflet of plasma membranes (24). The predicted topology of mouse podocin has the intracellular N- and C-terminal regions, a transmembrane domain, and the PHB domain, which unlike the PHB of flotillins, is not involved in hairpin formation (22). Flotillins form homo- and heterophillic interactions mediated *via* the C-terminal region conserved only within the flotillin family while in podocin both the N- and C-terminal domains are predicted to mediate homooligomerization (22, 24). As of now, nothing is known about the molecular organization of the podocin hairpin domain or whether podocin undergoes palmitoylation and/or myristoylation. The PHB domain of flotillins plays an essential role in endocytic trafficking of several receptors, including those activated by the Src family kinase Fyn (29). Nephrin is phosphorylated by Fyn, and the phosphorylation augments nephrin interaction with podocin and facilitates nephrin endocytosis *via* a clathrin-independent pathway, activating nephrin signaling (15, 30). By contrast, dephosphorylation of the nephrin-Y^1193^ increases nephrin endocytosis mediated by β-arrestin, decreases nephrin signaling, and impairs the slit diaphragm integrity (31). These data suggest that different endocytic pathways may play opposite roles in modulating nephrin function and that the endocytic itineraries of nephrin are regulated by the phosphorylation/dephosporylation cycle (see below).

PKC-α, stimulated by the high glucose concentration, induces nephrin endocytosis and leads to proteinuria while depletion of PKC-α stabilizes nephrin in diabetic nephropathy (32, 33). PKC-α phosphorylates two residues in the intracellular domain of nephrin, T^1120^ and T^1125^, and facilitates nephrin interaction with β-arrestin in murine podocytes (16). These data suggest that PKC-α promotes β-arrestin-mediated nephrin endocytosis, most likely *via* the clathrin-dependent pathway. Following clathrin-dependent endocytosis, receptors are transported either to the fast or slow recycling route, depending on the stability of interaction between the receptor and β-arrestin. In addition to regulating endocytosis, β-arrestin controls post-endocytic itineraries of several receptors. Nothing is currently known about post-endocytic sorting of nephrin. Understanding the structural basis and kinetics of binding between nephrin and β-arrestin may help to elucidate the fate of internalized nephrin, whether it undergoes lysosomal degradation or recycling to the plasma membrane or both.

CD2AP may control nephrin endocytic trafficking by regulating actin assembly and vesicle sorting. The importance of CD2AP in podocytes is demonstrated by glomerulosclerosis and foot process effacement, leading to renal failure in mice lacking CD2AP (34). CD2AP localizes in the slit diaphragm cytoplasmic region juxtaposed to the lipid raft membranes and co-localizes with the actin-related protein 2 (ARP2) and ARP3 (Arp2/3) and cortactin. CD2AP regulates endosomal sorting and vesicle trafficking by regulating actin assembly (35). Moreover, CD2AP associates with the dynamic actin pool and co-localizes with the endocytic adaptors Rab5, Rab4, the clathrin-dependent endocytic adaptor assembly polypeptide-2 complex (AP-2), and participates in formation of multivesicular bodies (35, 36). CD2AP interacts with podocin and nephrin, anchoring the proteins to the actin cytoskeleton (37, 38). CD2AP and nephrin interact with the p85 regulatory subunit of the phosphoinositide 3-OH kinase (PI3K), leading to its recruitment to the plasma membrane (39). PI3K plays an essential role in endocytic trafficking, including regulation of membrane lipid composition, release of clathrin-coated vesicles, and the Rab5 recruitment (40). The interactions of PI3K with nephrin, CD2AP, and other slit diaphragm proteins suggest that PI3K plays an important role in endocytic trafficking at the slit diaphragm (41).

CIN85/Ruk_L_ is a closely related homolog of CD2AP (42). In podocytes, CD2AP blocks CIN85/Ruk_L_ expression by sumoylation while CD2AP depletion increases CIN85/Ruk_L_ abundance and leads to CIN85/Ruk_L_-mediated nephrin ubiquitination and endocytosis (43, 44). CIN85/Ruk_L_ depletion has the opposite effect and preserves nephrin expression at the slit diaphragm membranes and reduces proteinuria in diabetic mice (45). Studies showed that unlike CD2AP, CIN85 does not contain an actin-binding domain (43, 46). Absence of CD2AP may facilitate nephrin interaction with CIN85, impair nephrin partitioning to lipid rafts, and induce nephrin endocytosis. However, another study has shown that CIN85 interacts directly with actin and together with CD2AP bundles actin filaments and modulates podocyte migration (47). Moreover, CIN85 clusters Src family kinases, providing a scaffold for Arp2/3-mediated actin assembly, and regulates cell polarization and motility. Expression of multiple CIN85 splice variants in different cell types may explain the reported difference of CIN85 function.

Notch activation alters foot process architecture in mature podocytes and induces proteinuria by stimulating the dynamin-dependent and lipid raft-independent nephrin endocytosis (17). The Wnt/planar cell polarity (PCP) pathway increases clathrin/β-arrestin-dependent nephrin endocytosis, and depletion of the PCP protein Vngl2 increases nephrin abundance at the cell surface and disturbs glomerular maturation (19).

The following protein networks have been shown to play a role in endocytic trafficking in podocytes, either directly or by regulating actin dynamics but their role in nephrin trafficking has not been demonstrated. α-Actinin-4 provides structural stability at the slit diaphragm by cross-linking and connecting actin filaments with nephrin and membrane associated guanylate kinase inverted (MAGI-1) (7, 48). The α*-actinin-4* gene mutations are associated with nephrotic syndrome called focal segmental glomerulosclerosis (49). Direct interactions between members of the multi-protein scaffold organized by α-actinin-4, MAGI-1, and the endocytic adaptor megalin indicate a role of the scaffold in endocytic trafficking at the slit diaphragm (50, 51). As a member of the cytoskeleton-associated recycling or transport (CART): an Hrs/actinin-4/BERP/myosin V, α-actinin-4 mediates constitutive recycling of transmembrane receptors *via* the rapid recycling route on actin filaments (52). The role of α-actinin-4 or other members of the CART complex in cargo recycling at the slit diaphragm remains unknown. Endocytic adaptors, synaptojamin 1, and endophilin 1–3 are critical for the foot process formation, while dynamin I and II play a role in the foot process maintenance (18).

## Structural Basis for Nephrin Endocytic Trafficking

A protein frequently utilizes different endocytic itineraries to diversify its function (1). Clathrin-dependent endocytosis is one of the most important internalization routes in eukaryotic cells (1). Endocytic adaptors recognize linear internalization signals located in the intracellular C-terminal tail domains of transmemembrane proteins and recruit these proteins as cargo to clathrin-coated pits, which are plasma membrane deformities coated with clathrin and clathrin-dependent endocytic adaptors. Subsequently, additional adaptors cleave off clathrin-coated pits and release them into the cell interior as clathrin-coated vesicles [reviewed in Ref (4)]. Clathrin-independent endocytosis includes a diverse group of internalization mechanisms sharing a requirement for free cholesterol, proteins, and lipids that reside in sphingolipid-rich lipid raft membranes (53).

Little is known about linear endocytic motifs in nephrin or other integral membrane proteins at the slit diaphragm. For example, the nephrin-Y^1193^ and subsequent amino acid residues D–E–V conform to a canonical, tyrosine-based endocytic signal of the YxxØ type, which is essential for clathrin-mediated endocytosis. Phosphorylation of the tyrosine residue inhibits the interaction of the YxxØ motif with the μ2 subunit of AP-2 and prevents endocytosis. By contrast, dephosphorylation of the tyrosine residue allows the YxxØ interaction with AP-2 and facilitates endocytosis. Thus, the phosphorylation state of the tyrosine residue serves as a regulatory switch controlling protein retention at the plasma membrane or its endocytosis *via* the clathrin-dependent pathway (54). Consistent with this model, Quack et al. demonstrated that β-arrestin mediates clathrin-dependent endocytosis of nephrin dephosphorylated at the conserved Y^1193^ residue (31). By contrast, phosphorylation of nephrin-Y^1193^ by Fyn augments nephrin interaction with podocin, prevents nephrin interaction with β-arrestin, attenuates β-arrestin-mediated nephrin endocytosis, and augments nephrin signaling (31). Moreover, the Fyn-mediated phosphorylation facilitates nephrin endocytosis *via* the clathrin-independent pathway (15, 30). These data demonstrate that the phosphorylation state of nephrin-Y^1193^ regulates nephrin signaling by directing nephrin to different endocytic pathways, either the clathrin-independent endocytosis to augment signaling or the clathrin-dependent endocytic to attenuate nephrin signaling. Although β-arrestin is known to interact predominantly with the clathrin-dependent adaptors, it was also found to modulate the clathrin-independent pathway by interacting with Arf6, and it may also utilize ubiquitination to inactivate receptor signaling (55, 56). At this time, the role of β-arrestin in nephrin ubuquitination or clathrin-independent internalization is unknown. Presence of several sequences conforming to the tyrosine-based endocytic motifs in nephrin cytoplasmic C-terminal domain suggests that regulation of nephrin endocytic itineraries is even more complex. The complexity is further increased by additional protein–protein interactions between nephrin, Fyn, and the actin cytoskeleton. Nephrin tyrosine phosphorylation is critical for recruitment of actin adaptors, such as p85/PI3K, Cas/Crk, and Nck, facilitating cytoskeletal dynamics in the podocyte foot processes (39, 41, 57–60). Nck facilitates Fyn-mediated nephrin phosphorylation, while Nck depletion leads to decreased nephrin tyrosine phosphorylation and foot process effacement (61, 62). Three conserved, Nck-binding Y–D–x–V motifs in the C-terminal nephrin tail, Y^1176^DEV, Y^1193^DEV, and Y^1217^DQV mediate these effects (63). Replacing the Y^1176^, Y^1193^, and Y^1217^ residues with the non-phosphorylated tyrosine mimic phenylalanine leads to proteinuria associated with foot process effacement, irregular thickening of the GBM, and dilated capillary loops in a mouse model (63).

## The Essential Role of Neph1 at the Mammalian Slit Diaphragm

Neph1, a transmembrane protein partially homologous to nephrin is another key component of the mammalian slit diaphragm (10, 64). Nephrotic syndrome resulting from mutations in the *neph1* gene in humans as well as severe proteinuria, foot process effacement, and early postnatal death in *neph1* KO-mice demonstrate its importance for the glomerular function (64). Although the extracellular domain of Neph1 can interact with nephrin, recent data show that such heterophilic interactions are rare while the hemophilic Neph1 and nephrin interactions are critical for the mammalian slit diaphragm architecture (10, 65). Similar to nephrin, the Neph1 cytoplasmic domain engages directly with podocin, anchoring the integral membrane proteins at the cell membrane and the scaffolding protein ZO-1 that connects with the cortical actin cytoskeleton (66, 67) (Figure 1). Upon signaling engagement, Neph1 and nephrin form a phosphorylation-dependent unit transmitting outside-in signaling through a multi-protein scaffold of junctional proteins to the podocyte actin network (68, 69). While the Fyn-mediated tyrosine phosphorylation of the Neph1 intracellular domain leads to recruitment of protein adaptor Grb2 necessary for the Neph1-mediated actin polymerization, tyrosine phosphorylation of nephrin intracellular domain leads to recruitment of Nck, necessary for nephrin-mediated actin polymerization (58, 69–71). Activation of the Neph1–nephrin functional complex occurs during junction formation and during podocyte injury, both requiring intense signaling and actin cytoskeleton reorganization (69). One study demonstrated that inhibiting Neph1 signaling in podocyte culture preserves podocyte architecture and function in the puromycin aminoglycoside (PAN) injury model, while maintaining high Neph1 levels protects podocytes from injury in *in vivo* zebrafish PAN and adriamycin injury models (72). Another group demonstrated that renal ischemia induces a rapid loss of Neph1 interaction with ZO-1 in an *in vivo* rat ischemic model (73). Induction of injury by ATP depletion in podocyte culture resulted in rapid loss of Neph1 and ZO-1 interaction and redistribution of both proteins from the cell membrane to the cytoplasm while recovery from the injury resulted in increased Neph1 tyrosine phosphorylation, restoring Neph1 and ZO-1 membrane localization and interaction (73). The structural basis for the Fyn-mediated Neph1–ZO-1 interaction essential for the podocyte function has been recently elucidated. The ZO-1 PDZ1 domain interacts directly with the PDZ-binding region in the C-terminus of Neph1 where the T^787^HV and L^761^T residues are critical for protein–protein binding and its stability (67, 74). The functional Neph1–nephrin unit also engages with the Par3–Par6–atypical protein kinase C (aPKC) complex to maintain a three-dimensional foot process architecture and integrity of the slit diaphragm (75). Neph1 may influence localization of other proteins in the foot process. For example, Neph1 interaction with the large-conductance Ca^2+^-activated (BK_Ca_) channel affects membrane localization of the channel (76). Data reviewed above attest to a dynamic role that Neph1 plays in the slit diaphragm remodeling. Yet, little is known about Neph1 trafficking itineraries. Emerging data demonstrate that Neph1 is recruited to the podocyte membrane by a motor protein Myo1c that interacts with Neph1 intracellular region directly *via* cargo-binding, C-terminal domain in an actin-dependent manner (20, 21). Moreover, Myo1c may control the cell membrane turnover of Neph1 (21). Myo1c also binds nephrin directly. However, by contrast to the effect on Neph1, Myo1c reduces nephrin localization at the podocyte membrane (20). It remain unknown how Myo1c exerts opposite effects on the membrane localization of Neph1 and nephrin.

## Summary

Recent data confirm that endocytic trafficking allows rapid regulation of the signaling strength and duration at the slit diaphragm. Identifying endocytic motifs of the integral membrane proteins and examining the structural basis of the protein–protein interactions that control endocytic trafficking may unravel novel mechanisms and enrich our understanding of the role of this essential biological function at the podocyte slit diaphragm, as well as teach about the mechanisms of nephrotic syndrome and measures to correct it.

## Author Contributions

AS-U reviewed the literature and wrote the manuscript.

## Conflict of Interest Statement

The author declares that the research was conducted in the absence of any commercial or financial relationships that could be construed as a potential conflict of interest.
